# Crop domestication disrupts intercropping benefits: A case study from barley–faba bean mixture under contrasting P inputs

**DOI:** 10.3389/fpls.2023.1153237

**Published:** 2023-03-08

**Authors:** Xiaoyan Tang, Zhengwu Wu, Siliu Hu, Guangdeng Chen, Rong Huang, Yingjie Wu, Bing Li, Qi Tao, Kemo Jin, Changquan Wang, Zhihui Wen

**Affiliations:** ^1^ College of Resources, Sichuan Agricultural University, Chengdu, China; ^2^ College of Resources and Environmental Sciences, National Academy of Agriculture Green Development, Key Laboratory of Plant-Soil Interactions, Ministry of Education, China Agricultural University, Beijing, China

**Keywords:** demestication, root functional traits, root plasticity, phosphorus uptake, species mixtures

## Abstract

How crop domestication mediates root functional traits and trait plasticity in response to neighboring plants is unclear, but it is important for selecting potential species to be grown together to facilitate P uptake. We grew two barley accessions representing a two-stage domestication process as a sole crop or mixed with faba bean under low and high P inputs. We analyzed six root functional traits associated with P acquisition and plant P uptake in five cropping treatments in two pot experiments. The spatial and temporal patterns of root acid phosphatase activity were characterized *in situ* with zymography at 7, 14, 21, and 28 days after sowing in a rhizobox. Under low P supply, wild barley had higher total root length (TRL), specific root length (SRL), and root branching intensity (RootBr) as well as higher activity of acid phosphatase (APase) in the rhizosphere, but lower root exudation of carboxylates and mycorrhizal colonization (MC), relative to domesticated barley. In response to neighboring faba bean, wild barley exhibited larger plasticity in all root morphological traits (TRL, SRL, and RootBr), while domesticated barley showed greater plasticity in root exudates of carboxylates and colonization by mycorrhiza. Wild barley with greater root morphology-related trait plasticity was a better match with faba bean than domesticated barley, indicated by higher P uptake benefits in wild barley/faba bean than domesticated barley/faba bean mixtures under low P supply. Our findings indicated that the domestication of barley disrupts the intercropping benefits with faba bean through the shifts of root morphological traits and their plasticity in barley. Such findings provide valuable information for barley genotype breeding and the selection of species combinations to enhance P uptake.

## 1 Introduction

Phosphorus (P) often limits plant growth, due to its low bioavailability and low mobility in most soils (Hinsinger, 2001). Under P deficiency, plants have evolved various strategies to improve P acquisition (Vance et al., 2003; Lambers et al., 2008). Diverse root functional traits underpin these strategies, including a) changes in root morphology and architecture to enhance soil exploration, such as higher specific root length (SRL) and longer and denser fine roots (Lynch, 2011; Richardson et al., 2011); b) increased release of root exudates to mobilize inorganic and organic P fractions (e.g., carboxylate or phosphatase as typical P-mobilizing exudates; Hinsinger et al., 2003; Wen et al., 2020); and c) strengthened symbiosis with mycorrhizal fungi to increase the soil volume accessible to the roots beyond the nutrient-depletion zone (Smith and Read, 2008). All the adjustments in these root functional traits contribute to increase plant P uptake, but they are also associated with significant carbon cost (Westoby and Wright, 2006; Liu et al., 2015). Thus, carbon cost may limit all these root functional traits being folded into one species or genotype because of inherent trade-offs between resource acquisition and resource conservation (Denison, 2012; Wen et al., 2019). Indeed, numerous lines of evidence showed that significant variation in root functional traits exists among and within plant species, resulting in a unique resource acquisition strategy and/or nutrient niche for a given species or genotype.

Combinations of species with different root traits and resource strategies could promote soil nutrient acquisition (e.g., P) and crop over-yielding (Hinsinger et al., 2011; Tang et al., 2021). The dissimilarity in root traits in consideration of the core elements reduces the intra-/interspecific competition through niche complementarity and promotes facilitation on P acquisition in species mixtures (Loreau et al., 2001; Li et al., 2014). It is generally accepted that species with P-mobilizing traits (e.g., root exudates for P mobilization) could facilitate the species without the traits when grown as mixtures, especially at low P condition (Yu et al., 2021; Yu et al., 2022). For example, P uptake benefits are significantly higher in cereal combination with faba bean and chickpea than with soybean based on the results of a meta-analysis (Tang et al., 2021). Overall, these results suggested that selecting the species with a good match in root functional traits for combinations is critical for the improvement of the P uptake benefits at the system level.

Plant domestication affected root functional traits through recurrent breeding selection to obtain traits that are regarded as favorable by humans (Eshed and Lippman, 2019; Preece and Peñuelas, 2020). Because of the high nutrient input and requirements of crop yields of modern agriculture, the outcome of domestication is crops obtained through resource acquisition strategies (Milla et al., 2014; Isaac et al., 2021). There is increasing evidence that crop domestication has profoundly altered root functional traits, such as SRL and root diameter, contributing to their successful adaption in agricultural conditions (e.g., N; Meyer et al., 2012). Aside from root morphological traits, root exudates between modern genotypes and their wild relatives have been reported (Gioia et al., 2015; Iannucci et al., 2017). Martín-Robles et al. (2018) reported that wild relatives of 27 crops benefited from arbuscular mycorrhizal fungi (AMF) colonization irrespective of P levels, while domesticated species relied on AMF only under P limitation conditions. However, how crop domestication affects the root trait syndromes and the potential trade-offs on root P acquisition strategies is still unclear.

Plant species can adjust their root functional traits (i.e., trait plasticity) to respond not only to the soil abiotic environment (e.g., P availability) but also to the presence of neighboring plants (Hodge, 2004; File et al., 2012). Root trait plasticity in response to neighbor species may also be shaped by the plastic capacity and particular neighbor species or genotypes (Abakumova et al., 2016; Henn et al., 2018). For example, Streit et al. (2019) reported that eight faba bean genotypes employed different root proliferation and distribution patterns in soil profile when wheat was grown as the neighboring plant. Lepik et al. (2012) found that the differential capacity of species in modulating their response to different neighboring genotypes is not uniform. This raises the question of whether and how crop domestication affects the root responses of the target plant (root trait covariation and plasticity) to neighboring plants. Specifically, do wild and domesticated genotypes respond to neighboring plants uniformly or differentially regarding root functional traits?

Barley (*Hordeum vulgare*) is the fourth most important cereal globally, with the longest course of domestication dating ~10,000 years (Bulgarelli et al., 2015). Growing mixtures of barley with legumes is a promising practice with efficient P uptake, although it depends on species combinations and P management (Darch et al., 2018). The P uptake benefits of barley–legume mixtures stem from the contrasting root exudation and morphological trait responses to P deficiency. Greater plasticity of specific traits could enhance the performance of species in mixtures (Yu et al., 2020). The authors found that species have greater root phenotypic plasticity in response to root exudates released by the P-mobilizing species under low P supply when grown in a mixture. The root traits of barley cultivars and legume species vary in response to different P inputs (Giles et al., 2017), but how the selected plants respond to their neighbors and the specific trade-offs in the expression of such root traits when grown in combinations was overlooked. Thus, we hypothesized that barley genotypes with greater root morphological trait plasticity to neighbors would have enhanced P uptake in barley–legume mixtures.

To address the above issues, we conducted a study with two genotypes of spring barley that represent two stages in the domestication process, namely, modern genotype Baudin (*H. vulgare* ssp. *vulgare*) and its wild relative CN4027 (*H. vulgare* ssp. *spontaneum*). Faba bean (*Vicia faba* L.) was selected to be grown in mixtures with barley under two contrasting P treatments (low P: severely limiting P without P addition; high P: adequate soil P availability). We aimed to 1) clarify how systemic changes and trade-offs affected root functional traits for P acquisition of the two genotypes representing different domestication stages; 2) evaluate how crop domestication shaped root morphological traits, exudation, and mycorrhizal colonization in response to faba bean grown as neighbors; and 3) assess whether domestication of barley affected root functional trait plasticity in governing P uptake benefits of the two mixtures.

## 2 Materials and methods

### 2.1 Plant materials and soil origin

The two barley genotypes were selected as test plant materials: spring barley modern genotype Baudin (*H. vulgare* ssp. *vulgare*) and its wild relative CN4027 (*H. vulgare* ssp. *spontaneum*). The P deficiency tolerance of wild barley (WB) was stronger than CN4027. The P acquisition efficiency in domesticated barley (DB) was bigger than in WB, while the P utilization efficiency in WB was bigger (Guo, 2017). The cultivar of faba bean (*Vicia faba* L.) was Qizi No. 7.

The tested soil was collected from the top 10 cm of a long-term P fertilizer field trial located in Beijing (39°59′N, 116°17′E) and sieved to 5 mm. The trial comprised two P regimes: P0 (low P, i.e., no P fertilization) and P80 (high P, i.e., 80 kg P ha^−1^ year^−1^). We collected the soil samples 20 years after the start of the field trial. The soil is a calcareous Cambisol (FAO-UNESCO, 1989). The low P soil had a pH (water) of 7.59, 7.6 g kg^−1^ of soil organic matter, 1.3 mg kg^−1^ of Olsen-P, 13.5 mg kg^−1^ of available N, and 70.0 mg kg^−1^ of NH_4_OAc-K. The high P soil properties were as follows: pH (water) 7.43, soil organic matter 13.5 g kg^−1^, Olsen-P 22.6 mg kg^−1^, available N 23.7 mg kg^−1^, and NH_4_OAC-K 98.8 mg kg^−1^.

### 2.2 Experimental setup

#### 2.2.1 Experiment 1

A pot experiment with five cropping treatments under two contrasted P levels was conducted to investigate how the root traits of the wild and domesticated barley respond to faba bean growing as a neighbor plant (**Supplementary Figures 1A, C**). The cropping treatments included WB, DB, and faba bean (FB) grown alone and two barley genotypes mixed with faba bean (WB/FB and DB/FB) in three replicates (30 pots in total). Each pot was filled with 2.5 kg of pre-prepared soil. When grown alone, barley was sown at four seeds per pot and faba bean was sown at two seeds per pot. To maintain the principle of substitution, the density of each species in the mixture was only half that of the sole crops. All the pots were arranged in a completely randomized design, with weekly randomization during the experiment.

#### 2.2.2 Experiment 2

A rhizobox experiment was conducted to investigate rhizosphere acid phosphatase of barley in different genotypes grown alone and with faba bean. The design of the 30-cm × 30-cm × 1.5-cm Perspex boxes with a removable side (**Supplementary Figure 1B**) allowed access to the roots to observe the spatial and temporal patterns of acid phosphatase with zymography (methods modified from Spohn and Kuzyakov, 2013). The soil properties and weight, choices of barley and faba bean, and cropping pattern in Exp. 2 were the same as in Exp. 1 (**Supplementary Figure 1A**), with five replicates (50 rhizoboxes in total). Each rhizobox was inclined at a 45° angle to encourage root growth over the soil surface. The water content in the rhizoboxes was maintained at 60% of field water-holding capacity throughout the experiment by weighing the rhizoboxes weekly.

To ensure that the soil nutrients can meet the needs of plant growth, the basic elements were added as a nutrient solution (mg kg^−1^) for both experiments: N-CO(NH_2_)_2_ 200, K-K_2_SO_4_ 100, Ca-CaCl_2_·2H_2_O 200, Mg-MgSO_4_·7H_2_O 50, Fe-EDTA-FeNa 5, Mn-MnSO_4_·4H_2_O 5, Zn-ZnSO_4_·7H_2_O 5, Cu-CuSO_4_·5H_2_O 5, B-H_3_BO_3_ 0.68, and Mo-Na_2_MoO_4_·5H_2_O 0.12. No additional sources of phosphorus were applied. Both experiments were conducted simultaneously from 15 January to 16 February 2022 in a glasshouse at Sichuan Agricultural University (30°57′N, 104°06′E). The temperature range was 25°C–30°C during the day and 18°C–22°C at night, and relative humidity was 60%–80%.

### 2.3 Harvest and measurements

Plants were harvested 30 days after sowing in Exp. 1. We collected all visible roots in each pot and carefully distinguished them for different species in the plant mixtures. For soil samples, we collected both the rhizosphere and bulk soil according to the definition and methods of Hinsinger (2001).

We measured seven root functional traits (**Supplementary Table 1**) associated with P acquisition and P mobilization. Root morphological traits included total root length (TRL), root branching intensity (RootBr), specific root length (SRL), and average root diameter (RootDiam). Root functional traits for P mobilization included the amounts of carboxylates and acid phosphatase activity in the rhizosphere (APase). Root colonization by AMF (MC) was also determined.

#### 2.3.1 Root morphological traits

The root samples were washed with deionized water and kept at 4°C for analysis. TRL and average RootDiam were analyzed with the software WinRHIZO 2009 (Regent Instruments Inc., Quebec, QC, Canada), based on the scanned images of the whole root system of each plant using a root scanner (ScanMaker i800 Plus, Microtek, USA). More than 10 intact root samples composed of first and second order roots were chosen randomly for RootBr measurements (Freschet and Roumet, 2017). The details of measuring and calculation of the root branching intensity are described by Wen et al. (2019) and Kong et al. (2014). SRL was calculated as the ratio of root length of the whole root system and its dry mass.

#### 2.3.2 Root exudation traits

Roots with rhizosphere were then transferred to a vial containing 50 ml of 0.2 mМ CaCl_2_ and gently shaken to determine carboxylate and acid phosphatase exudation (Pearse et al., 2007). The root exudates of carboxylates and APase activity were analyzed following the methods of Neumann (2006) and Shen et al. (2003).

#### 2.3.3 Root colonization by AMF

Thirty root segments (1-cm-long) were randomly selected and cleared with 10% (m/v) KOH solution in a 90°C water bath for 50 min, acidified with 2% (v/v) HCl for 5 min at room temperature, and then stained with Trypan blue reagent in a 90°C water bath for 30 min (Philips & Hayman, 1970). Then, the root segments were observed under a light microscope (Vierheilig et al., 1998). The colonization of AMF (%) was calculated according to the method of Trouvelot et al. (1985).

#### 2.3.4 Plant biomass and nutrient analysis

The shoots and roots were oven-dried at 105°C for 30 min and then at 65°C to a constant weight and weighed for mass determination. The dried plant samples were crushed and sieved, digested by H_2_SO_4_-H_2_O_2_, and the P content was determined by vanadium-molybdenum yellow colorimetry (Johnson and Ulrich, 1959).

#### 2.3.5 Soil P availability analyses

Both the rhizosphere and bulk soil subsamples were air-dried and ground to <2 mm. Soil available P was extracted with NaHCO_3_ (0.5 M, pH = 8.5) and then acidified with 37% (v/v) HCl to precipitate organic matter. The phosphorus content in the extract was determined by the malachite green method at 630 nm wavelength (Ohno and Zibilske, 1991).

#### 2.3.6 Soil zymography

Zymography, as an *in-situ* non-destructive technique, was performed to test the spatial and temporal patterns of acid phosphatase (ACP) as affected by genotypes and plant interactions at 7, 14, 21, and 28 days after plant sowing in the rhizobox experiment. The measurement of soil enzyme activity was at a consistent location throughout the study, as we labeled the tested root zone between plants. The fluorescently labeled substrate 4-methylumbelliferyl-phosphate (MUF) (Sigma-Aldrich, Germany) (Spohn and Kuzyakov, 2013) was dissolved in MES buffer (C_6_H_13_NO_4_SNa_0.5_) (Sigma-Aldrich, Germany) to a concentration of 10 mM (Koch et al., 2007). The membrane (0.45 μm pore size, 20 cm diameter) was completely saturated with the substrate solution and then dried in a shade. The rhizoboxes were opened, and the soil surface was covered with the membrane in the root observation area for 1 h (Razavi et al., 2016). Afterward, the membrane was placed in a dark box, the UV lamp was turned on, and fluorescent photographs were taken at 8 s exposure; all filming conditions were consistent during the experiment. Root-associated area analysis (as described by Spohn and Kuzyakov, 2013) was used to measure the root-associated enzyme activity. The details of image analysis were described by Schofield et al. (2019).

A calibration curve was established to relate the enzyme activity to the fluorescence intensity of the images. The calibration curves were prepared by soaking the membranes (2 × 2 cm) in the increased MUF concentrations (0, 0.01, 0.05, 0.1, 0.5, 1, 3, and 6 mM), using the same procedure as in the experiment. The amount of MUF absorbed on the membrane was calculated based on the concentration and volume of the solution (Liu et al., 2017).

### 2.4 Statistical analysis

For Exp. 1, we first tested whether P levels, cropping system (sole *vs*. mixture), and barley genotype (wild *vs*. domesticated) affected root functional traits using linear models. Then, we tested whether root functional traits were influenced by barley genotypes within the P level with Student’s *t*-test (*p* ≤ 0.05). To characterize the plasticity of seven root functional traits of the two genotypes of barley (wild *vs*. domesticated) in response to faba bean grown as a neighboring plant, we calculated the response ratio (RR) of each root trait (Valladares et al., 2006; Li et al., 2019). The RR was defined as the extent of root response grown as a sole crop and mixtures, using the following equation:


$$\text{RR}=\sum \frac{\left(R_{i'j'}-R_{ij}\right)}{\left(R_{i'j'}+R_{ij}\right)}/n$$

Where *R_i’ j’_* and *R_ij_* are the root traits of species grown as a mixture and sole crop, respectively. The *i′* and *i* represent the given species grown as a mixture and sole crop. The *j′* and *j* are two randomly selected individuals from three replicates of the same genotype in the mixture and sole crop, respectively. The *n* is the number of *R_i’ j’_*-*R_ij_* values. The difference (*p* ≤ 0.05) in RR values between the two genotypes of barely grown with faba bean was tested with Student’s *t*-test.

In Exp. 2, differences in the hotspot of APase activity between two P level treatments and between wild and domesticated barley grown as sole or mixture with faba bean were tested with the analysis of variance (ANOVA).

To test whether domestication of barley influenced plant growth and P uptake benefits in the barley and faba bean mixture, we calculated land equivalent ratio (LER) and net effect (NE) in plant biomass (LER_B_ and NE_B_) and P uptake (NE_B_ and NE_P_) and tested the differences between the two mixtures using Student’s *t*-test. The equations and the definition of LER and NE calculation are shown in the **Supplementary Information** (**S1-Eq. 1, 2**).

To clarify how domestication of barley determined the effectiveness of root traits in plant growth and P uptake, principal component analysis (PCA) was performed to determine the multivariate ordination of seven plant traits of two barley genotypes in two mixtures and under two P conditions. Pearson’s correlation analysis was conducted to test the relationships among root functional traits. The analyses were performed with Origin (OriginPro, Version 2023. OriginLab Corporation, Northampton, MA, USA).

## 3 Results

### 3.1 Plant growth and P uptake benefits in the species mixture

**Figure 1:**
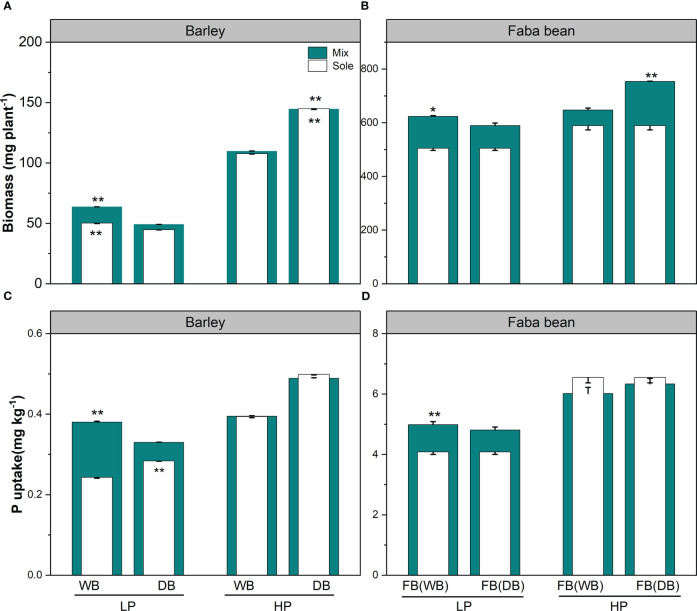
Biomass **(A, B)** and P uptake **(C, D)** for barley and faba bean grown as sole crops and mixtures under contrasting P conditions. The open and closed bars represent plants grown as soles and mixtures, respectively. Values are the means of three replicates. The error bars represent the standard deviation (SD) of the mean. Within the P treatment, asterisks denote a significant difference between the wild and domesticated barley or faba bean grown with them (significance: *, *p*< 0.05; **, *p*< 0.01; ***, *p*< 0.001; ns, not significant). FB(WB), faba bean in FB/WB; FB(DB), faba bean in FB/DB; Sole, WB, DB, and FB grown alone; Mix, faba bean grown with WB and faba bean grown with DB.

At the harvest, WB had significantly higher biomass than DB grown either as a sole crop or in mixture under low P (**Figure 1A**). In contrast, DB had better plant performance than WB both in sole crop and mixture at high P condition (**Figure 1A**). Faba bean grown with WB had 1.23-fold higher biomass than grown with DB under low P, while faba bean with DB had higher biomass than grown with WB under high P (**Figure 1B**). As for P uptake, DB had significantly higher uptake than WB when grown as sole, while WB had 1.15-fold higher P uptake than DB in the mixtures under low P (**Figure 1C**). We observed higher P uptake for faba bean grown with WB than with DB under low P (**Figure 1D**).

In the low P treatment, we found benefits in the plant biomass (LER_B_, NE_B_) and P uptake (LER_P_, NE_P_) for both WB/faba bean and DB/faba bean, whereby LER_B_ and LER_P_ were higher than 1 and NE_B_ and NE_P_ were higher than 0 (**Table 1** and **Supplementary Table 1**). The WB/faba bean had better performance in biomass and P uptake than DB/faba bean (1.1-fold for each LER_B_ and LER_P_ and 44.9 and 0.21 mg pot^−1^ for NE_B_ and NE_P_, respectively). Under P sufficiency, no plant biomass and P uptake benefits were observed for the two species mixtures (**Table 1** and **Supplementary Table 1**).

**Table 1:** Land equivalent ratio (LER), net effect (NE) based on plant biomass and P uptake, and competitive balance index (CBI) of barley (wild barley or domesticated barley)/faba bean intercropping under two contrasting P inputs.

Phosphorus level	Cropping system	LER_B_	LER_P_	NE_B_ (mg pot^−1^)	NE_P_ (mg pot^−1^)
LP	WB/FB	1.43 a	1.29 a	323.8 a	0.96 a
	DB/FB	1.30 b	1.17 b	278.9 b	0.75 b
HP	WB/FB	0.92 A	0.96 A	−245.4 A	−0.53 A
	DB/FB	0.89 A	0.97 A	−198.7 B	−0.97 B

Values represent the means of three replicates. For a given P fertilizer level, different letters indicate a significant difference between WB/FB and DB/FB (p< 0.05).

LER_B_, LER based on plant biomass; LER_P_, LER based on P uptake; NE_B_, NE based on biomass; NE_P_, NE based on P uptake; LP, low P; HP, high P; WB, wild barley; DB, domesticated barley; FB, faba bean.

### 3.2 Root functional traits in response to neighbors under two P levels

**Figure 2:**
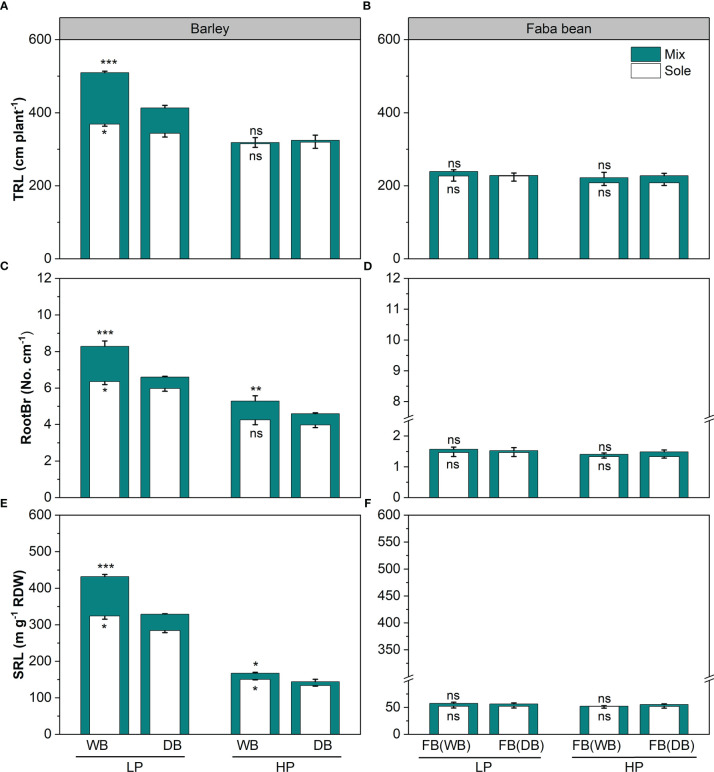
Total root length (TRL; **A, B**), root branching intensity (RootBr; **C, D**), and specific root length (SRL; **E, F**) for barley and faba bean grown as sole crops and mixtures under contrasting P conditions. The open and closed bars represent plants grown as soles and mixtures, respectively. Values are the means of three replicates. The error bars represent the standard deviation (SD) of the mean. Within the P treatment, asterisks denote a significant difference between the wild and domesticated barley or faba bean grown with them (significance: *, *p*< 0.05; **, *p*< 0.01; ***, *p*< 0.001; ns, not significant). FB(WB), faba bean in FB/WB; FB(DB), faba bean in FB/DB; Sole, WB, DB, and FB grown alone; Mix, faba bean grown with WB and faba bean grown with DB.

**Figure 3:**
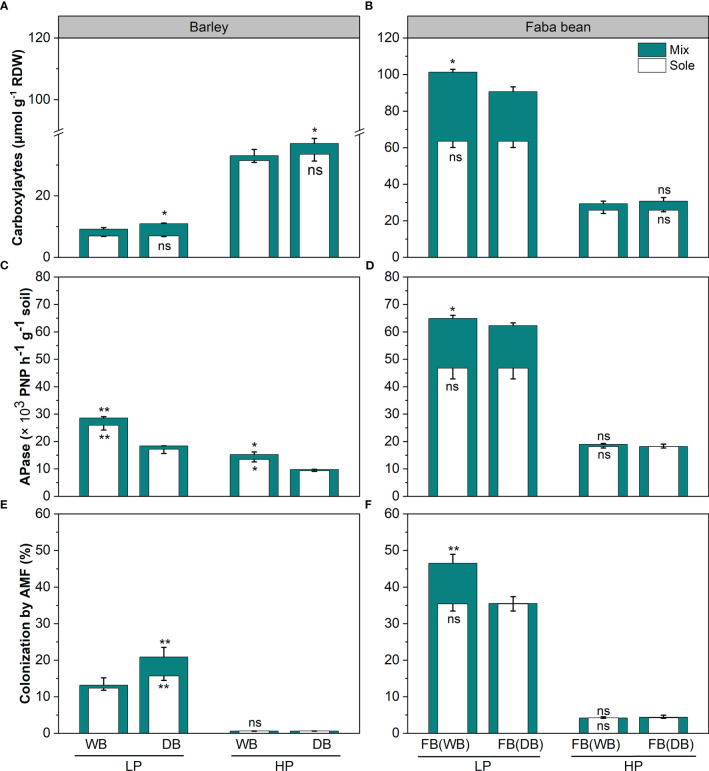
The amount of rhizosphere carboxylates per dry weight **(A, B)**, activity of acid phosphatases **(C, D)**, and mycorrhizal colonization (MC; **E, F**) for barley and faba bean as sole crops and mixtures under contrasting P conditions. The open and closed bars represent plants grown as sole crops and mixtures. Values are the means of three replicates. The error bars represent the standard deviation (SD) of the mean. Within the P treatment, asterisks denote a significant difference between the wild and domesticated barley or faba bean grown with them (significant level: *, *p*< 0.05; **, *p*< 0.01; ***, *p*< 0.001; ns, not significant). FB(WB), faba bean in FB/WB; FB(DB), faba bean in FB/DB; Sole, WB, DB, and FB grown alone; Mix, FB grown with WB and FB grown with DB.

The genotypes of barley (wild *vs*. domesticated) and phosphorus (P) levels significantly affected the root functional traits of barley (*p<* 0.01; **Table 2** and **Figures 2**, **3**). Under P deficiency, WB had significantly higher TRL, SRL, and RootBr than DB (**Figures 2A, C, E**). The rhizosphere exudation of APase was 1.45-fold higher in WB than DB at low P (**Figure 2C**). When P supply was sufficient, faba bean had significantly higher SRL (**Figure 2E**) and rhizosphere APase (**Figure 3C**), but lower rhizosphere carboxylates than DB (**Figure 3A**). The sufficient soil P availability significantly increased the amount of rhizosphere carboxylates in barley (**Figure 3A**) but decreased the remaining five root functional traits of barley and faba bean grown either as a sole crop or as mixtures (**Supplementary Table 3** and **Figures 2**, **3**).

**Table 2:** The *p*-values of the linear model analyzing six root functional traits.

Species	Barley					
Cropping pattern	Sole barley, mixture					
P levels	LP, HP					
Parameter	TRL	RootBr	SRL	Carb	APase	MC
Barley genotype (G)	0.297	0.199	0.305	0.552	**0.007**	0.417
Cropping pattern (CP)	**0.038**	0.053	0.314	0.505	0.593	0.662
P levels (P)	**<0.001**	**<0.001**	**<0.001**	**<0.001**	**<0.001**	**<0.001**
G × CP	0.118	0.102	0.537	0.857	0.057	0.822
G × P	**<0.001**	**<0.001**	**<0.001**	**<0.001**	**<0.001**	**<0.001**
CP × P	**<0.001**	**<0.001**	**<0.001**	**<0.001**	**<0.001**	**0.001**
G × CP × P	**<0.001**	**<0.001**	**<0.001**	**<0.001**	**<0.001**	**<0.001**
Species	Faba bean					
Cropping pattern	Sole faba bean, mixture					
P levels	LP, HP					
Parameter	TRL	RootBr	SRL	Carb	APase	MC
Barley genotype (G)	0.189	0.169	0.855	**<0.001**	**<0.001**	**<0.001**
P levels (P)	**0.085**	**0.019**	0.932	0.517	0.736	0.853
G × P	**0.043**	**0.041**	**0.033**	**<0.0001**	**<0.001**	**<0.001**
Cropping pattern	Intercrop					
Species	Barley, faba bean					
P levels	LP, HP					
Parameter	TRL	RootBr	SRL	Carb	APase	MC
Barley genotype (G)	0.539	0.595	0.593	0.950	0.587	0.909
P levels (P)	0.074	0.231	**0.041**	0.131	**<0.001**	**<0.001**
Species (S)	**<0.001**	**<0.001**	**<0.001**	**0.001**	**0.004**	**0.038**
G × P	0.258	0.631	0.2145	0.513	**0.003**	**<0.001**
P × S	**<0.001**	**<0.001**	**<0.001**	**<0.001**	**0.001**	**<0.001**
G × P × S	**<0.001**	**<0.001**	**<0.001**	**<0.001**	**<0.001**	**0.001**

The subsets were created for the cropping pattern (sole barley, mixture, sole faba bean), phosphorus levels (LP and HP for low and high, respectively), and species (barley, faba bean). Given are the p-values for the factors of barley genotype, cropping pattern, P levels, species, and their interactions. Bold p-values indicate the significant factors and interactions at p ≤ 0.05.

Root functional traits: TRL, total root length; RootBr, root branching intensity; SRL, specific root length; Carb, the total amounts of carboxylates in the rhizosphere; APase, acid phosphatase activity in the rhizosphere; MC, colonization by arbuscular mycorrhizal fungi.

**Figure 4:**
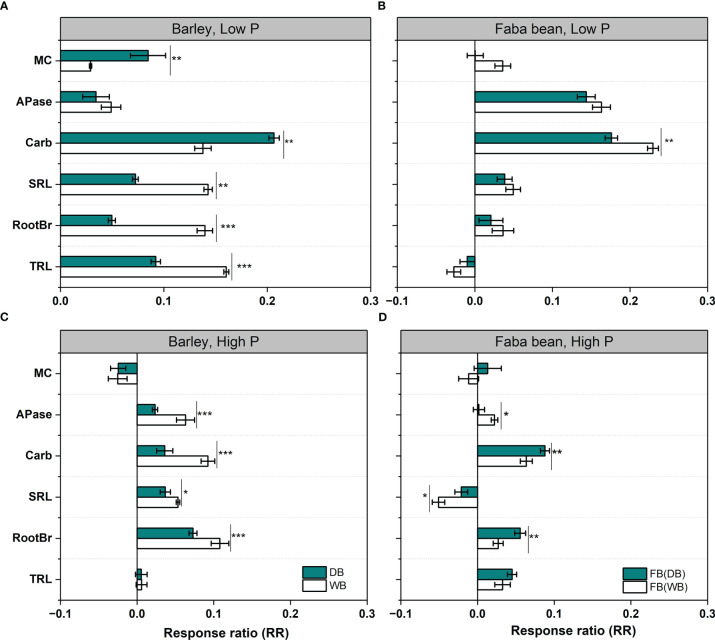
The response ratio (RR) of six root functional traits of wild and domesticated barley **(A, C)** and faba bean mixed with barley **(B, D)** under low P **(A, B)** and high P **(C, D)**. Asterisks denote a significant difference in the given functional traits between the wild and domesticated barley or faba bean grown with them in the given functional traits based on Student’s *t*-test (significant level: *, p< 0.05; **, p<0.01; ***, p< 0.001; ns, not significant). Data are the means + standard deviation (*n* = 9). Trait abbreviations: MC, colonization by arbuscular mycorrhizal fungi; APase, acid phosphatase activity; SRL, specific root length; RootBr, root branching intensity; TRL, total root length.

The response pattern of root functional traits to neighbors could be assessed by RR (**Figure 4**). Both WB and DB showed a positive response to faba bean grown as a neighboring plant in root morphology, root exudation, and mycorrhizal colonization under low P treatment (**Figure 4A**). The values of RR for DB were close to zero, except for carboxylates (**Figure 4A**). The domestication of barley significantly affected the magnitude of RR to faba bean: WB had a significantly higher increase in RTL, RootBr, and SRL than DB (1.3-, 2.6-, and 2.0-fold, respectively), whereas DB had significantly increased higher exudation (1.45-fold) and mycorrhizal colonization (2.75-fold) than WB. The response patterns indicated that WB had a high plasticity of root morphological traits, whereas DB had a high plasticity in root exudation and mycorrhizal colonization in response to faba bean. For faba bean, the only negative RR value was for TRL, and the RR values of RootBr, SRL, and MC approached zero (**Figure 4B**). We assumed that faba bean had low plasticity in root morphological traits and mycorrhizal colonization. When faba bean was mixed with WB, it had a positive RR value of carboxylate exudation (significantly higher than that in the faba bean/DB mixture) (**Figure 4B**). The RR values of six functional traits (except mycorrhizal colonization) for barley and the SRL for faba bean were positive (but close to zero), indicating low plasticity of root traits under sufficient P supply (**Figures 4C, D** **)**.

**Figure 5:**
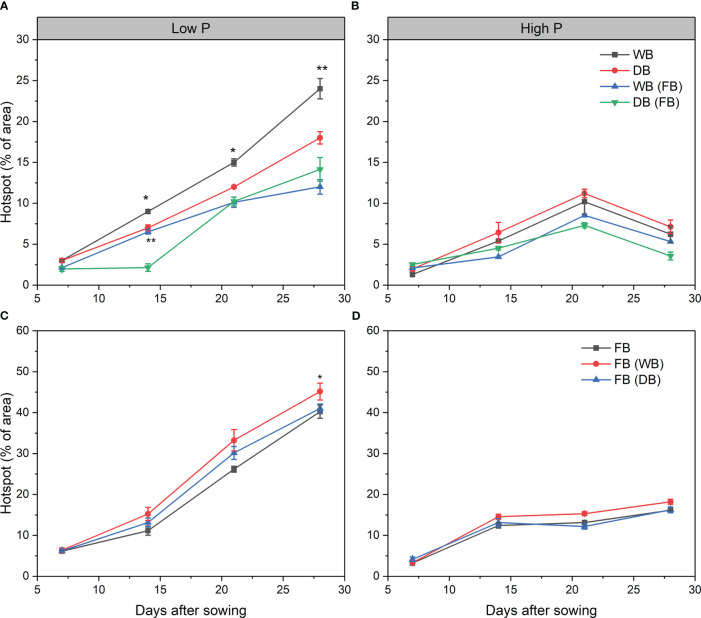
Enzymatic hotspot areas of acid phosphatase activities in soil under low **(A, C)** and high P **(B, D)** inputs. Significant differences between two barley genotypes or faba bean grown with them are indicated by asterisks (significance: * *p*< 0.05, ** *p*< 0.01, *** *p*< 0.001). Significant differences between sampling times are shown in **Supplementary Table 2**. FB(WB), faba bean in FB/WB; FB(DB), faba bean in FB/DB; Sole, WB, DB, and FB grown alone; Mix, FB grown with WB and FB grown with DB.

The hotspots of APase activity were the highest and nearest to the root axis, as indicated by the brightest area (**Supplementary Figures 3**, **4**). At high P, the APase activity pattern remained constant, with only a slight increase at 21 days across all the treatments (**Figures 5B, D**; **Supplementary Figure 4** and **Supplementary Table 3**). There was no significant difference in APase activity between the different genotypes of barley and faba bean grown as a sole crop or as mixtures under sufficient P (**Figures 5B, D**). By contrast, the hotspots of APase were more dispersed and increased with time both around the root axis and away from the root at low P (**Figures 5A, C**; **Supplementary Figure 4** and **Supplementary Table 2**). For barley, the hotspots of APase were significantly higher for WB than DB at 14, 21, and 28 days (1.38-, 1.48-, and 2.0-fold, respectively) (**Figure 5A**). However, this difference disappeared in the mixtures with faba bean at 14 days (**Figure 4A**). The APase activity pattern of faba bean was similar regardless of whether it was grown with WB or DB from 7 to 21 days. On day 28, faba bean grown with DB had slightly but significantly increased APase activity relative to faba bean grown with WB (*p*< 0.05) (**Figure 5C**).

### 3.3 Multivariate coordination

**Figure 6:**
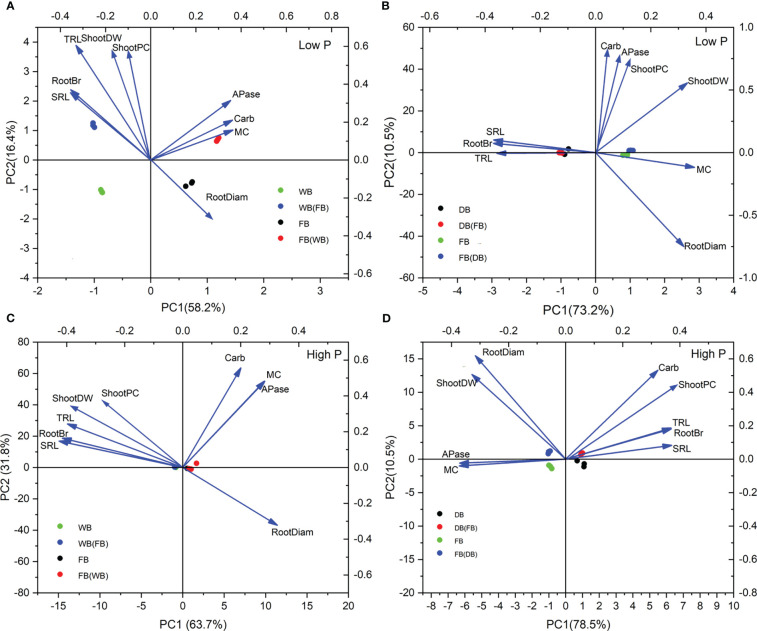
The first two principal components from PCA based on shoot growth, shoot P uptake, and seven root functional traits for two cropping mixtures under low P **(A, B)** and high P **(C, D)** treatments.

As for WB/faba bean at low P, PCA based on nine plant traits explained 94.6% of the variation. The first principal component (PC1) was primarily determined by AMF colonization, two P-mobilizing exudation traits, and shoot P content, accounting for 78.2% of the total variation. The second principal component (PC2) accounted for 16.4% of the total variation and was determined mainly by shoot biomass, TRL, root branching intensity, and SRL (**Figure 6A**). WB and faba bean grown alone or as a mixture were clustered and distinctly separated, indicating dissimilar patterns in root functional traits in response to low P among species and cropping patterns. By contrast, we only observed DB and faba bean distinctly separated into two groups at low P (**Figure 6B**). **Figure 6B** shows that TRL, RootBr, and SRL scored high on the PC1 (73.2%) and the root exudate-related parameters and shoot P content accounted for the PC2 (10.5%; **Figure 6B**). Barley was in the direction of TRL, RootBr, and SRL, whereas faba bean was clustered and scattered in the direction of the root exudation trait for the two combinations under low P conditions.

In WB/faba bean at high P, PC1 dominantly comprised the root morphological trait parameters, accounting for 63.7% of the variability; PC2 represented 31.8% of the variance and mainly included AMF symbiosis, two P-mobilizing exudation traits, and shoot P content (**Figure 6C**). In DB/faba bean at high P, PC1 and PC2 represented 78.5% and 10.5%, respectively. The mycorrhizal colonization and root morphological traits were mainly on the first axis, whereas carboxylate exudation, RootDiam, shoot P content, and biomass scored high on the second axis (**Figure 6D**).

## 4 Discussion

### 4.1 Root trait variation between wild and domesticated genotypes

The shifts in root functional traits with domestication were illustrated when we compared the root traits between wild and domesticated barley (**Figures 2**–**4**). Specifically, wild barley was characterized by higher TRL, SRL, RootBr, and APase activity under low P conditions (**Figures 1**, **2**). The contrasting patterns in root functional traits represent different P acquisition strategies between WB and DB, which may result from their distinct environment of habitat/growth. Wild relatives usually grow under conditions with nutrient (including P) constraints and/or with high heterogeneous nutrient distribution (Lambers et al., 2008), while the modern genotypes were commonly selected in soils rich in available nutrients (Martín-Robles et al., 2019; Isaac et al., 2021). Thus, wild relatives with higher SRL and root branching intensity (i.e., competitive root morphological traits) may enable their survival in the wild conditions by a fast resource foraging strategy (Kembel et al., 2008). In addition, differing from intensive agricultural landscapes with high chemical fertilizer inputs, soil nutrients in natural habitats of wild relatives were rich in organic forms (e.g., P), and sparingly soluble organic P cannot be absorbed by plants without hydrolyzing to soluble inorganic phosphate (Richardson et al., 2011); thus, increased root exudation of acid phosphatases may contribute to enhance the mobilization of soil organic P for wild barley.

Both WB and DB in this study benefited from AMF colonization grown as a sole crop under low P, which is in line with the study of Martín-Robles et al. (2018) who found that wild progenitors relied on AMF regardless of deficient or sufficient P in comparison with 27 crop species. However, the domestication of barley is more mycorrhizal-responsive than wild genotypes, which were characterized by a fine root system. The higher mycorrhizal colonization and root carboxylate exudation under low P conditions in DB indicated that the lack of functional traits (i.e., rapid root growth and APase exudation) caused shifts to other traits (carboxylate exudation and mycorrhizal colonization in this study) with complementary functions under low P conditions. This supported the finding that domestication might have led to the evolution toward resource-acquisitive strategies under higher resource input (Martín-Robles et al., 2018). The strength and direction of the response of mycorrhizal colonization to soil P availability were similar for the two barley genotypes. These traits may, however, have high carbon cost, and their development may be downregulated if the respective function is not required (Raven et al., 2018). Under adequate soil P availability, the importance of mycorrhizal colonization was reduced in domesticated barley. Our result showed that both the wild and domesticated barley, based on inherent trade-offs of root functional traits, develop a complementary capacity to acquire P present at different availabilities. However, the effect of domestication on root traits shifts and the trade-off is diverse.

### 4.2 Domestication of barley mediated root trait plasticity in response to neighbors

Our hypothesis that domestication shapes the plasticity of root traits has been proven, indicated by barley variation in terms of plasticity of root traits in response to faba bean grown as a neighboring plant under low P conditions (**Figure 3**). WB exhibited greater root plasticity in root morphological traits, while DB had a greater increase in root exudation of carboxylates and mycorrhizal colonization (**Figure 3A**). Except for APase activity, the remaining five root functional traits of barley, namely, TRL, RootBr, SRL, carboxylates, and MC, displayed appreciable positive responses to neighboring faba bean (**Figure 3A**). These changes in root morphological traits in WB might improve the ability of the roots to forage for bioavailable P in the soil and reduce energy use in construction consumption, which help wild barley to adapt to soil containing a low concentration of readily exchangeable inorganic phosphate (Lynch, 2019). In our study, we found that WB had higher P uptake than DB with neighboring faba bean, which increased P availability *via* root exudation (**Figure 1B** and **Supplementary Figure 1**). We assumed that the variation in root traits in response to the neighbor was due to inherent plasticity, and this study proved that it weakened in the course of domestication and affected P uptake. Understanding root plasticity is important for plant nutrient acquisition either grown as a sole crop or in mixtures at the species or genotypic level, due to the soil nutrient heterogeneity (Wang et al., 2020). Therefore, more studies are required to reveal the potential genetic basis of the plasticity of barley root during domestication for breeding to enhance nutrient use efficiency in the modern agricultural ecosystem.

Faba bean exhibited larger plasticity in P-mobilizing exudation than in root morphology when grown in mixtures (**Figure 3B**). This is in line with previous studies on 20 chickpea genotypes (Wen et al., 2020). Wild barley, as a neighboring plant (Callaway et al., 2003; Abakumova et al., 2016; Garlick et al., 2021), induced greater carboxylate exudation of faba bean than DB. However, when the P supply was sufficient, root trait plasticity in response to neighboring plants can be ignored for both WB and DB, with RR values near zero (**Figures 3C, D**). This indicated that nutrient availability mediated the magnitude of root trait plasticity in response to neighboring plants.

Our hypothesis that WB and DB grown with neighboring faba bean would have different spatial and temporal patterns of APase activity was not supported in the present study. We found the hotspots of APase activity increased steadily with time and in a similar pattern across all species (**Figure 4**). Hence, it seemed that plant–plant interaction did not change the temporal and spatial dynamics of APase activity. Schofield et al. (2019) also found that the temporal dynamics of soil enzyme activity was influenced only by the intracultivar plant–plant competition. Thus, we surmised that the inherent stability of the species was greater than the influence from neighboring species.

### 4.3 Phosphorus uptake benefits in two mixtures as influenced by the domestication of barley

We hypothesized that barley/faba bean intercrop only has plant growth and P uptake benefits at low P conditions. Substantial enhancement of resource use efficiency of the barley/faba bean intercropping system regardless of the genotype of barley was indicated by the land equivalent ratio for biomass (LER_B_ = 1.43 ± 0.05; 1.30 ± 0.01) and the net effect for biomass (NE_B_ = 323.8 ± 18.2; 278.9 ± 3.2) for WB/faba bean and DB/faba bean, respectively, at low P (**Table 1**). This is consistent with previous studies, where barley/legume intercropping showed advantages in P uptake, but only under P limitation (Darch et al., 2018; Strydhorst et al., 2008; Tang et al., 2021). This indicated that P fertilizer input levels played crucial roles in P uptake benefits by mixtures through positive rhizosphere interactions.

Based on the mechanisms of enhanced P uptake in cereal/legume intercrop (Li et al., 2014; Yu et al., 2022), our hypothesis was that faba bean, as a species with stronger P mobilization activity, grown with greater root morphological plasticity barely could exhibit higher P uptake benefits. The hypothesis was supported by WB/faba bean exhibiting significantly higher benefits in P uptake and plant biomass than DB/faba bean (**Table 1**). The relatively higher P uptake benefits of WB/faba bean can be attributed to two reasons. Firstly, greater positive root responses in TRL, RootBr, and SRL of wild barley enabled its higher P uptake, indicated by a positive correlation between shoot P content and root morphological traits in the WB/faba bean combination (**Figure 6A**). Secondly, higher P availability in the rhizosphere of the WB/faba bean mixture amplified such benefits (**Supplementary Figure 2A**), which was attributed to more exudation of carboxylates by faba bean grown with wild barley than with domesticated barley (**Figure 6B**; Hinsinger et al., 2011). Such plastic response of root traits in the WB/faba bean mixture is complementary to accessing more P under P deficiency. This is in line with previous studies in maize/faba bean (Zhang et al., 2016; Zhang et al., 2020) and steppe species combinations (Yu et al., 2020). The authors found that maize proliferated roots in the proximity of faba bean roots that had greater P availability in the rhizosphere (Callaway and Li, 2019; Zhang et al., 2020), resulting in higher P uptake by the maize/faba bean mixture. As for the DB/faba bean combination, shoot P content and biomass relied predominantly on the root exudation traits of faba bean rather than that of barley (**Figure 6B**). This indicated that greater root plasticity in exudates of carboxylates was functionally redundant for the mixture when the neighboring faba bean could release greater carboxylates than barley. In addition, the greater root plasticity in carboxylate and mycorrhizal colonization was associated with higher carbon consumption compared with the root morphological changes (Wagg et al., 2015). Thus, non-P-mobilizing species with greater plasticity in nutrient-foraging traits are of great importance when grown with P-mobilizing species to enhance P uptake. Therefore, aside from the species-specific P acquisition strategies, it is also important to consider root functional plasticity, especially for non-P-mobilizing species, to achieve P facilitation in the species mixtures under low P supply.

Furthermore, domesticated barley and wild relative showed contrasting mixture effects with neighboring plants (i.e., faba bean), suggesting that crop domestication disrupts intercropping benefits. The general conclusion on how domestication affects yield and P uptake benefits in intercrops requires testing using a wider range of crop species and genotypes. Recent studies have proposed a “back to the roots” or “a return to the wild” framework to explore the microbiome assembly traits or root exudate traits of wild relatives of crop species which have been undermined during plant domestication (Pérez-Jaramillo et al., 2016; Preece and Peñuelas, 2020). The intercropping system, as a promising option to enhance resource use efficiency in intensive agriculture (Tilman, 2020), root morphological functional traits, and plasticity, should not be neglected in the framework of barley breeding for intercropping with legumes to enhance P uptake.

## 5 Conclusion

We focused on how the domestication of barley affects the root functional traits in response to contrasting soil P availability and neighboring faba bean. WB was characterized by root morphological traits with low carbon cost, including higher TRL, SRL, and RootBr to get access to P economically and efficiently. Furthermore, in response to neighboring faba bean, WB exhibited a large plasticity in root morphological traits (including TRL, SRL, and RootBr), whereas DB showed greater plasticity in root exudates of carboxylates and colonization by AMF. Finally, faba bean facilitated more P uptake when intercropped with wild barley, due to its larger root morphological plasticity than domesticated barley under low P supply. Overall, our study presented here can be a valuable addition to the understanding of domestication effects on root trait inherent trade-offs and its plasticity response to neighbors when selecting species combinations for enhanced P acquisition.

## Data availability statement

The original contributions presented in the study are included in the article/**Supplementary Material**. Further inquiries can be directed to the corresponding author.

## Author contributions

XYT, ZWW and GDC designed the study. ZWW, SLH, RH, YJW and BL performed the experiment and collected the data. XYT and ZWW analyzed the data. QT, CQW, ZHW and JMK discussed the data and led the writing of the manuscript. All authors contributed to the article and approved the submitted version.
