# Supplementary material for: Crop domestication disrupts intercropping benefits: A case study from barley–faba bean mixture under contrasting P inputs
Source: Front Plant Sci. 2023 Mar 8;14:1153237. doi: 10.3389/fpls.2023.1153237 (PMC10030718; doi:10.3389/fpls.2023.1153237)
Supplement: Supplementary file 1 [file DataSheet_1.docx]

Supplementary Material

**Table of contents**

| **S1** | Experimental design and equipment | p. 2 |
| --- | --- | --- |
| **S2** | Equations for LER and NE calculation | p. 3 |
| **S3** | Supplementary results | p. 4 |

**S1 Experimental design and equipment**


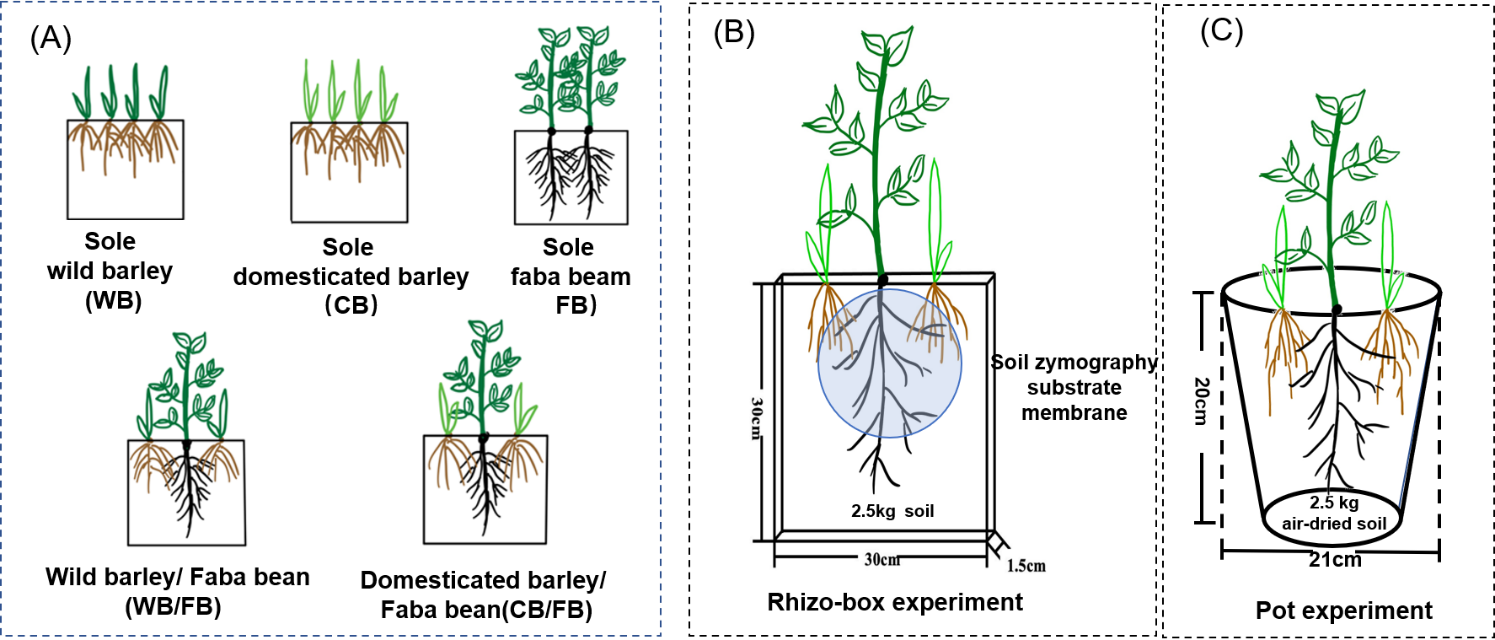


**Supplementary Figure 1** A schematic diagram of different cropping treatments (A) in rhizo-box (B) and pot (C)

**S2 Equations 1 and 2 for LER and NE calculation**

To evaluate the effect of different intercropping system on plant biomass and phosphorus uptake, the land equivalent ratio (LER) was used to measure the relative advantage of the intercropping system in biomass or P uptake (LER_B_ or LER_P_):

$LER=\frac{Y_{1}}{M_{1}}+\frac{Y_{2}}{M_{2}}$ (1)

Y_1_ and Y_2_ is the biomass or phosphorus uptake of intercropped barley and faba bean, respectively. M_1_ and M_2_ is the biomass or phosphorus uptake of sole barley and faba bean. When LER>1, it means that the intercropping system has a relative advantage in biomass or phosphorus uptake compared with the sole system. When LER<1, it means that the intercropping system has no relative advantage in biomass or phosphorus uptake compared with sole system (Tang et al., 2021).

To get the absolute value of intercropping effect on on crop biomass and P uptake, we used net effect on biomass (NE_B_) and P uptake (NE_P_) as evaluation parameters, both of which were calculated as the observed value of crop yield (biomass) or P uptake (Y_obs_) minus the expected value of crop yield (biomass) or P uptake (Y_exp_):

$\mathrm{NE}_{Y}=Y_{\mathrm{obs}}-Y_{\exp}=\sum Y_{i}-\sum P_{i}\times M_{i}$ (2)

The observed value of crop yield is the sum of crop biomass or P uptake in intercropping, whereas the expected value is the sum of crop biomass or P uptake in monoculture multiplied by their corresponding proportion (P) in intercropping. When NE>0, the intercropping system had a net biomass or P uptake increase effect, and when NE<0, the intercropping system had no net biomass or P uptake increase effect (Li et al., 2020).

**S3 Supplementary results**

Table 1 The abbreviations, descriptions and units of each root functional trait measured in the experiment

| Parameter | Abbreviation | Description | Units |
| --- | --- | --- | --- |
| **Root morphological traits (P acquisition)** | | | |
| Average root diameter | RootDiam | Average diameter of combined first- and second-order order roots | mm |
| Total root length | TRL | Total length of the whole root system | cm |
| Specific root length | SRL | Length per unit dry mass of combined first two order roots | m g^-1^ |
| Root branching intensity | RootBr | Number of first-order roots per  unit length of second-order  roots | No. cm^-1^ |
| **Root exudates traits (P mobilization)** | | |  |
| Rhizosphere root exudates carboxylates in rhizosphere | Carboxylates/Carb | The total amount of carboxylates in rhizosphere soil from whole root systems | μmol g^-1^ RDW |
| Acid phosphatase activity in rhizosphere | APase | Rate of substrate p-nitrophenylphosphate (NPP) hydrolyzed per minute at 25^o^C in sodium acetate buffer (pH 5.2) | ×10^3^ PNP h^-1^ g^-1^ soil |
| **Mycorrhizal traits** |  |  |  |
| Colonization by arbuscular mycorrhizal fungi (AMF) | MC | Percentage of absorptive root length colonized by arbuscules, vesicles or coils | % |

Supplementary Table 2 Results of ANOVA analyses on LER_B_, LER_P_, NE_B_ and NE_P_ (*p-values*). The p-values of P levels, genotypes and P levels **×** genotypes come from the two-way (P levels **×** species) ANOVA analyses. P levels: Low P *versus* High P; Genotypes: wild barley versus domesticated barley

|  | P levels  (n=16) | Genotypes  (n=16) | P levels **×** Genotypes  (n=16) |
| --- | --- | --- | --- |
| LER_B_ | <0.001 | <0.05 | <0.05 |
| LER_P_ | <0.001 | <0.05 | <0.05 |
| NE_B_ | <0.001 | <0.01 | <0.01 |
| NE_P_ | <0.001 | <0.01 | <0.01 |

Supplementary Table 3 Different letters indicate the significant differences in acid phosphatase activity between sampling times for species grown as sole crop and mix.

| P level | Treatment | 7 day | 14 d | 21 d | 28 d |
| --- | --- | --- | --- | --- | --- |
| Low P | WB | d | c | b | a |
|  | DB | d | c | b | a |
|  | WB (FB) | d | c | b | a |
|  | DW (FB) | b | b | ab | a |
|  | FB | d | c | b | a |
|  | FB (WB) | d | c | b | a |
|  | FB (DW) | d | c | b | a |
| High P | WB | c | b | a | b |
|  | DB | c | b | a | b |
|  | WB (FB) | c | b | a | b |
|  | DW (FB) | c | b | a | b |
|  | FB | b | a | a | a |
|  | FB (WB) | b | a | a | a |
|  | FB (DW) | b | a | a | a |


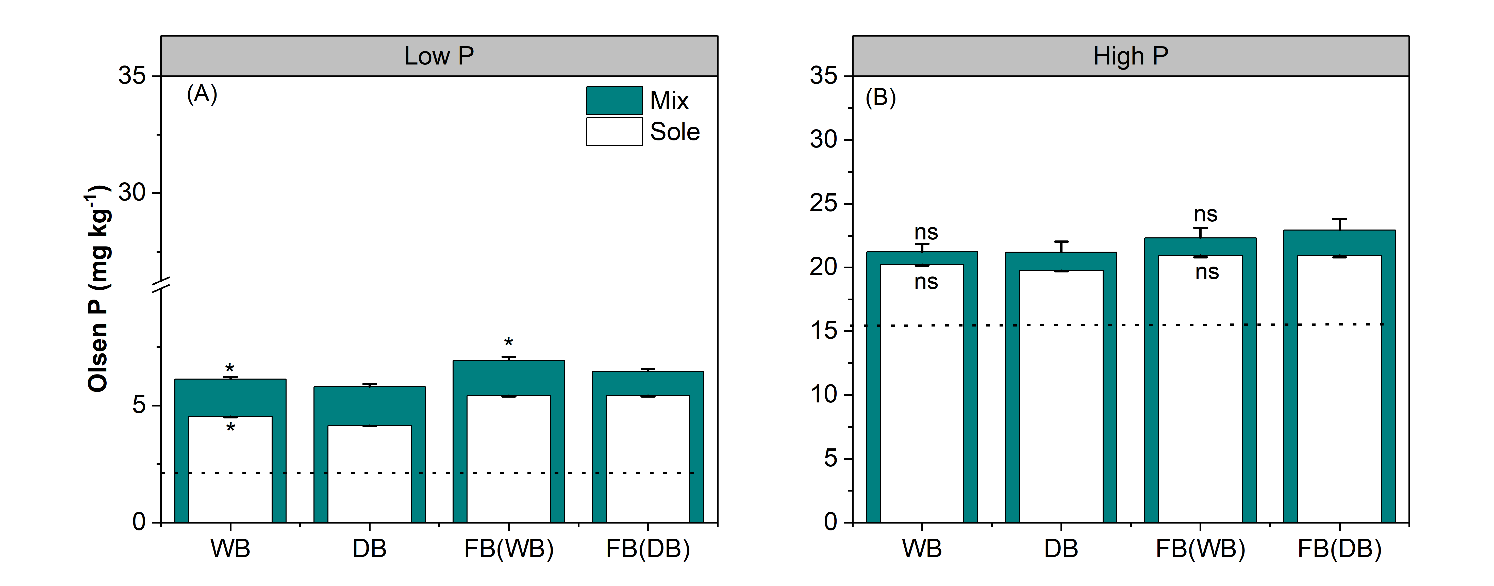


Figure 2 Inorganic phosphorus in Olsen extracts (Olsen P) of soil in which barley and faba bean were grown as sole crops or mixtures at low P (A) and high P (B). Values are means of four replicates. The error bars represent the standard deviation of the mean. Dotted lines were the average values of Olsen P in the bulk soil across all the treatments under low P and high P. Asterisks denote a significant difference between the wild and domesticated barley or faba bean grown with them (significance: *p < 0.05, **p < 0.01, ***p < 0.001, ns, not significant). FB(WB), faba bean in FB/WB; FB(DB), faba bean in FB/DB; Sole, WB, DB, FB grown alone; Mix, FB grown with WB and FB grown with DB.


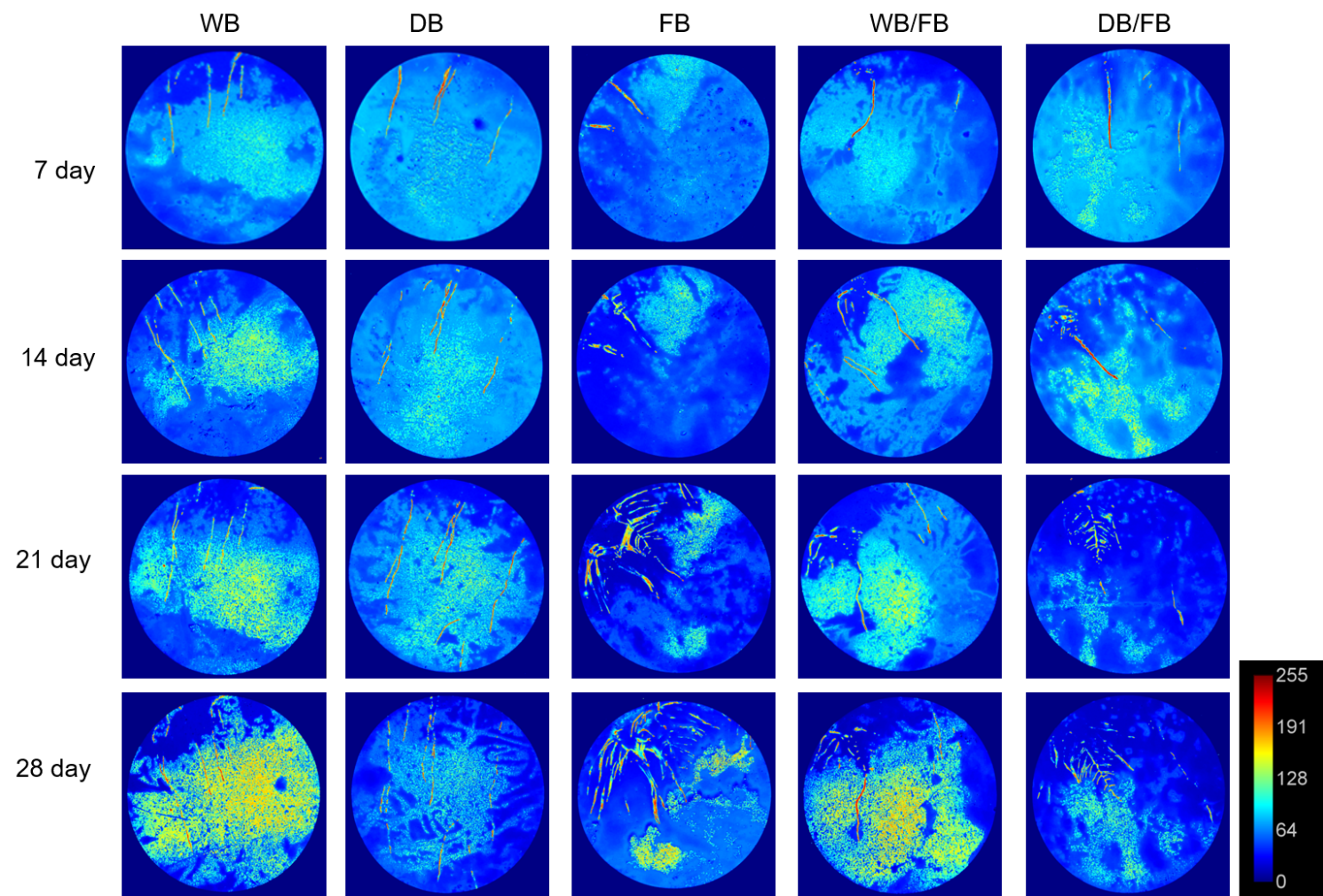


**Figure 3** Soil zymograms images for acid phosphatase activities around barely and faba bean root sampled from plants grown as sole crop and mix at low P (n=5) at 7, 14, 21 and 28 days after plant sowing.


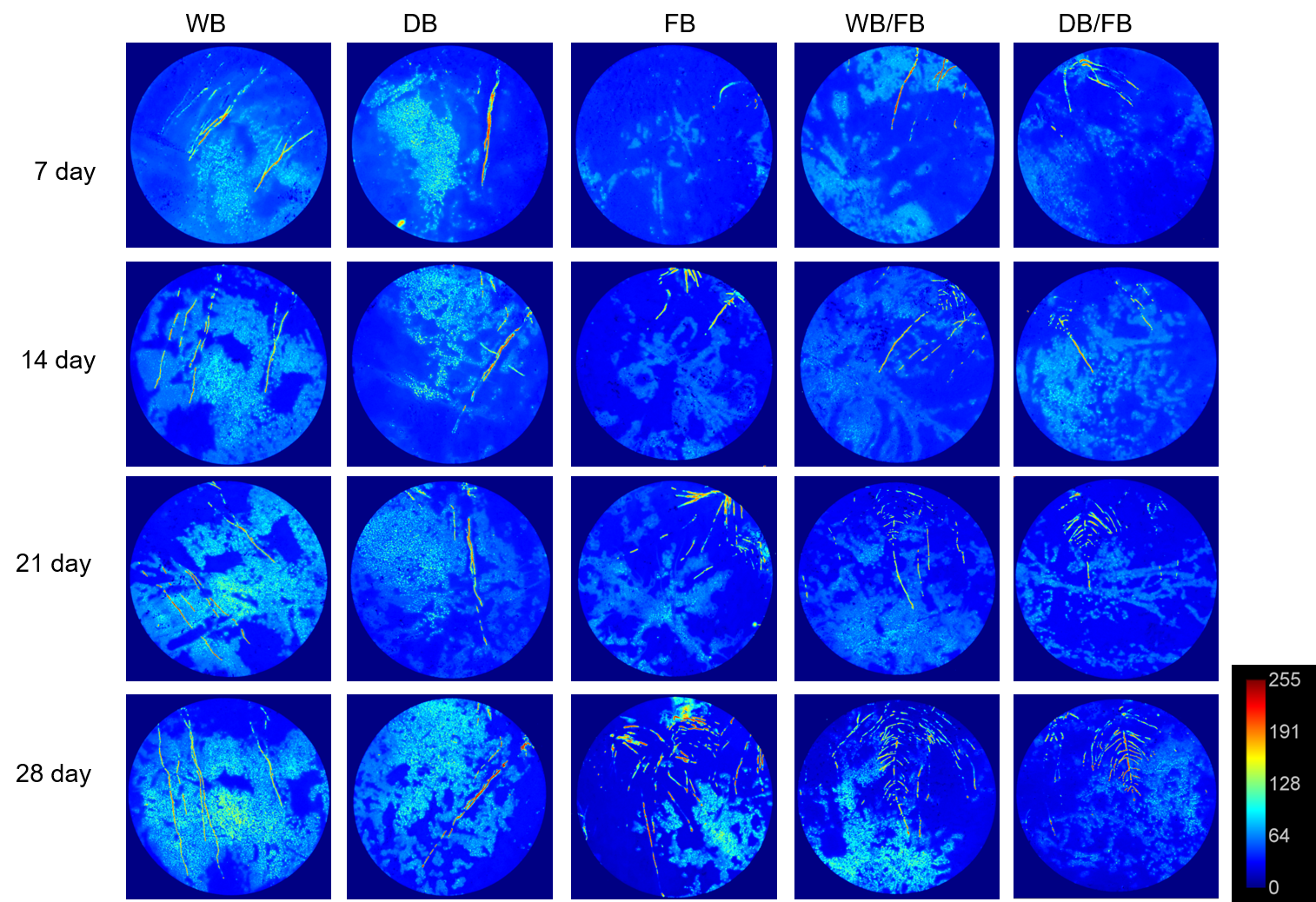


**Figure 4** Soil zymograms images for acid phosphatase activities around barely and faba bean root sampled from plants grown as sole crop and mix at high P (n=5) at 7, 14, 21 and 28 days after plant sowing.





**Figure 5** Heat map of Pearson’s correlation coefficients with original data among plant root traits under low P (A) and high P (B).
